# Saturated long-chain fatty acid-producing bacteria contribute to enhanced colonic motility in rats

**DOI:** 10.1186/s40168-018-0492-6

**Published:** 2018-06-14

**Authors:** Ling Zhao, Yufen Huang, Lin Lu, Wei Yang, Tao Huang, Zesi Lin, Chengyuan Lin, Hiuyee Kwan, Hoi Leong Xavier Wong, Yang Chen, Silong Sun, Xuefeng Xie, Xiaodong Fang, Huanming Yang, Jian Wang, Lixin Zhu, Zhaoxiang Bian

**Affiliations:** 1 Chinese Medicine Clinical Study Center, Jockey Club School of Chinese Medicine, Hong Kong Baptist University, Kowloon Tong, Hong Kong SAR China; 2 BGI Genomics, BGI-Shenzhen, Shenzhen, China; 3 Preparatory Office of Shenzhen-Melbourne Institute of Life Sciences and Bioengineering, Guangzhou University of Chinese Medicine, Guangzhou, China; 4 YMU-HKBU Joint Laboratory of Traditional Natural Medicine, Yunnan Minzu University, Kunming, China; 5 The Second Affiliated Hospital of Guangzhou University of Chinese Medicine, Guangzhou, China; 6 BGI-Shenzhen, Shenzhen, China; 7 Digestive Diseases and Nutrition Center, Department of Pediatrics, The State University of New York at Buffalo, 3435 Main Street, 422BRB, Buffalo, NY 14214 USA

**Keywords:** Gastrointestinal motility disorder, Gut microbiota, Neonatal maternal separation, Saturated long-chain fatty acids

## Abstract

**Background:**

The gut microbiota is closely associated with gastrointestinal (GI) motility disorder, but the mechanism(s) by which bacteria interact with and affect host GI motility remains unclear. In this study, through using metabolomic and metagenomic analyses, an animal model of neonatal maternal separation (NMS) characterized by accelerated colonic motility and gut dysbiosis was used to investigate the mechanism underlying microbiota-driven motility dysfunction.

**Results:**

An excess of intracolonic saturated long-chain fatty acids (SLCFAs) was associated with enhanced bowel motility in NMS rats. Heptadecanoic acid (C17:0) and stearic acid (C18:0), as the most abundant odd- and even-numbered carbon SLCFAs in the colon lumen, can promote rat colonic muscle contraction and increase stool frequency. Increase of SLCFAs was positively correlated with elevated abundances of *Prevotella*, *Lactobacillus*, and *Alistipes*. Functional annotation found that the level of bacterial LCFA biosynthesis was highly enriched in NMS group. Essential synthetic genes *Fabs* were largely identified from the genera *Prevotella*, *Lactobacillus*, and *Alistipes*. Pseudo germ-free (GF) rats receiving fecal microbiota from NMS donors exhibited increased defecation frequency and upregulated bacterial production of intracolonic SLCFAs. Modulation of gut dysbiosis by neomycin effectively attenuated GI motility and reduced bacterial SLCFA generation in the colon lumen of NMS rats.

**Conclusions:**

These findings reveal a previously unknown relationship between gut bacteria, intracolonic SLCFAs, and host GI motility, suggesting the importance of SLCFA-producing bacteria in GI motility disorders. Further exploration of this relationship could lead to a precise medication targeting the gut microbiota for treating GI motility disorders.

**Electronic supplementary material:**

The online version of this article (10.1186/s40168-018-0492-6) contains supplementary material, which is available to authorized users.

## Background

Disordered gastrointestinal (GI) motility is one major symptom frequently presented in patients suffering with functional GI disorders (FGIDs) and other bowel diseases [1, 2]. Chronic or recurrent episodes of dysregulated GI motility severely impact patients’ quality of life [3]. The underlying mechanisms of disordered GI motility are multifaceted. Previous studies have revealed contributions of immune activation, ionic channels and neurohumoral dysregulation to GI motor dysfunction [4–6]. However, current etiological understanding of disordered GI motility is incomplete, which limits the development of personalized and precisely effective medicine for GI motility disorders.

The gut microbiota is thought to be an important factor associated with disordered GI motility [7]. For example, patients with irritable bowel syndrome (IBS), especially for diarrhea-predominant IBS (IBS-D), showed a lower diversity and a higher instability of gut microbial community [8, 9]. Altered composition of the gut microbiota was also found in children and adults with chronic functional constipation [10–12]. Regulation of the gut microbiota by probiotics can improve bowel movement frequency in up to 70% of functionally constipated patients [13]. Transplantation of fecal microbiota from IBS-D suffers significantly accelerated colonic transit in germ-free (GF) mice [14]. GF mice colonized with fecal microbiota from patients with slow transit constipation exhibit lower fecal frequency, delayed GI transit time, and weaker spontaneous contractions of colonic smooth muscle [15]. These results point directly to a conclusion that the gut microbiota serves a pivotal role in GI motility disorder. But the mechanism by which microbiota interact with and affect host GI motility remains unclear.

It has been proposed that the effect of the gut microbiota on host GI motility partly derives from release of bacterial end-products of fermentation or molecules derived from host and gut microbiota co-metabolism [16, 17]. One study found that microbial products, such as lipopolysaccharides (LPS), can regulate GI motility through activating toll-like receptor 4 (TLR4) signaling that increases survival of nitrergic neurons [18]. Short-chain fatty acids and lactic acids, produced from bacterial fermentation of dietary fiber and resistant starch, can regulate intestinal motor action, probably through regulating peptide YY secretion and cholinergic neurons [19]. In another study, GF mice displayed accelerated GI motility after colonization with spore-forming microbes, the metabolites of which could stimulate enteric serotonin release [20]. Therefore, gut microbiota-derived substances appear to be the link between gut microbiota and host GI motility.

A rodent model of neonatal maternal separation (NMS) is a well-established model characterized by long-term colonic dysfunction [21]. Our previous study found that colon tissue isolated from adult NMS rats showed a higher level of muscle amplitude [22]. Such enhanced colonic motility is involved in upregulation of L-type calcium channels in colonic smooth muscle cells [22] and increased serotonin production from colonic enterochromaffin cells (ECs) [23]. Meanwhile, gut microbial disturbance is also reported in both mice and rats subjected to NMS [24, 25], but the association of changed gut microbiota with colonic dysmotility in an NMS model is yet to be investigated.

In this study with an NMS rat model, metabolic profiling of feces and luminal contents of the GI tract of experimental animals was preformed to identify featured microbiota-derived metabolites. A group of saturated long-chain fatty acids (SLCFAs) was remarkably increased in the colon lumen and feces of NMS rats. After confirming their stimulatory effects on colon motility, function-based metagenomic analysis was done and found hyperactive synthesis of SLCFAs by the gut microbiota of NMS rats. The relationship between SLCFA-producing bacteria and host colon motility were further clarified by transplanting fecal microbiota from NMS donors into pseudo germ-free (GF) rats and modulating gut microbiota in NMS rats by neomycin intervention. Based on these experiments, we aimed to clarify the linkage between gut microbiota and host colonic motility phenotype and to determine which bacteria are the primary agents manipulating host GI colonic dysmotility.

## Results

### Excess intracolonic SLCFAs are associated with GI motility in NMS model

**Fig. 1:**
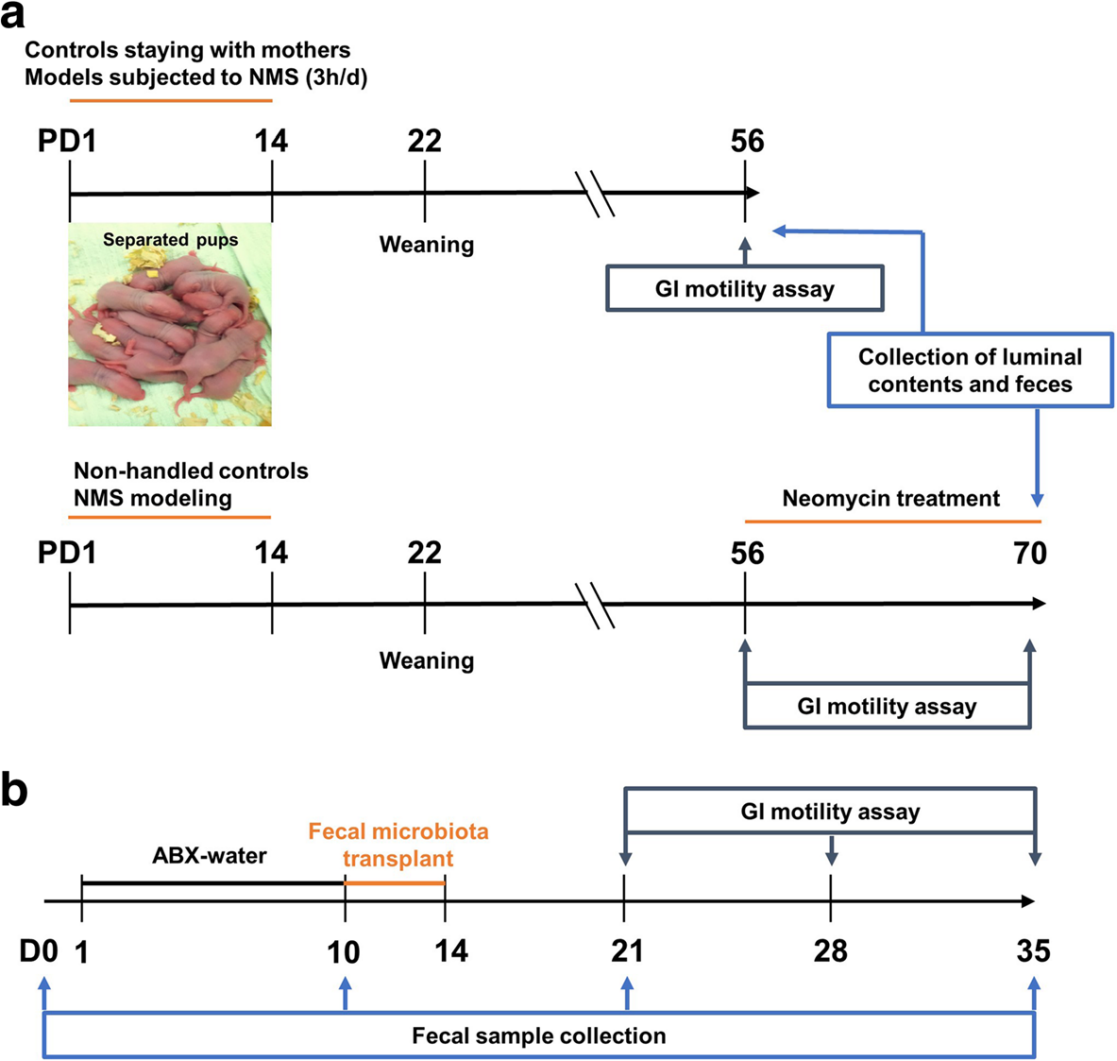
The detail procedures for animal experiments in neonatal maternal separation (NMS) model (*n* = 8/group) and pseudo germ-free (GF) model (*n* = 6/group). **a** Neonatal pups were separated with mothers for 3 h daily (9 a.m. to 12 a.m) from postnatal days (PD) 1 to 14, while other pups staying with mother are controls. The GI motility of rats was elevated by defecation frequency and gut transit time at adulthood. Feces were collected from both groups for metabolomic and metagenomic analysis. In addition, 150 mg/kg of neomycin was twice daily treated to NMS rats from PD 56 to 70 for confirming the relationship between SLCFA-produced bacteria and host bowel motility. **b** The pseudo GF rats model was induced by intaking water of antibiotic cocktails (ABX) for ten consecutive days. Fecal microbiota from NMS or control donors were prepared as PBS suspension for oral gavage to GF rats from day 10 to 14. The stool frequency was monitored weekly 1 week after fecal microbial transplantation. Fecal samples were collected from day 0, 10, 21 and 35 for observing dynamic changes of fecal microbiota at duration of the experiment

**Fig. 2:**
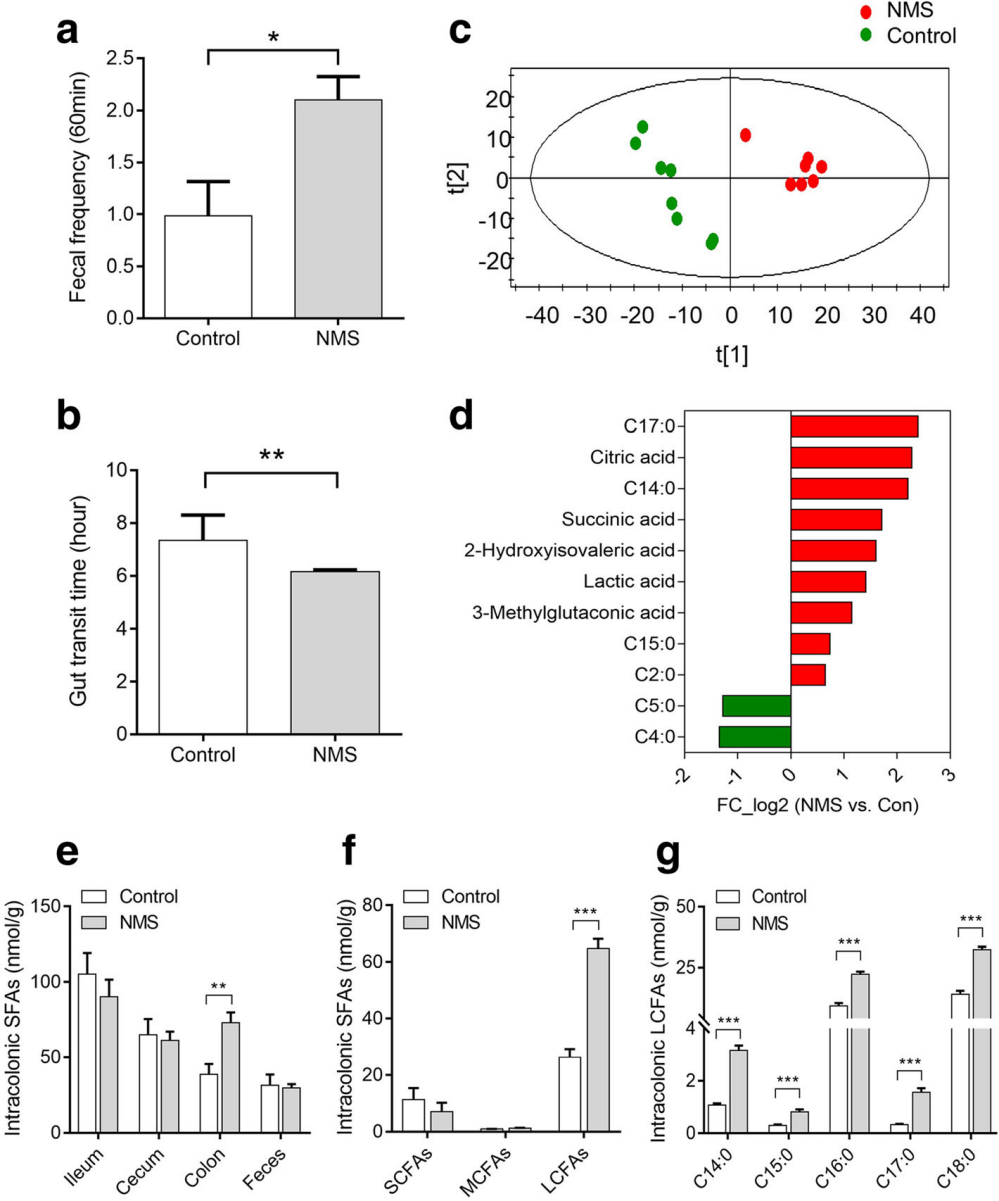
NMS rats are characterized by enhanced gut motility and upregulated intracolonic saturated long-chain fatty acids (SLCFAs) relative to controls (*n* = 8/group). **a** Accumulation of fecal pellet output within 60 min. **b** The gut transit time measured by oral administration of carmine red marker. **c** The scatter plot of fecal volatile organic compounds (VOCs) based on partial least squares discriminant analysis (PLS-DA). *X* and *Y* axes (t1 and t2) indicate the first two discriminating vectors, which respectively explain 33.5 and 16.6% of variation in the dataset. **d** Alteration of acidic substances derived from microbial metabolism in NMS rats. **e** The level of total saturated fatty acids (SFAs) per gram of luminal contents collected from ileum, cecum, proximal colon and feces. **f** The total levels of saturated short-chain, medium-chain, and long-chain fatty acid per gram of colonic contents in rats. **g** The level of individual SLCFAs per gram of colonic contents in rats. C2:0, acetic acid; C4:0, butyric acid; C5:0, valeric acid; C14:0, tetradecanoic acid; C15:0, pentadecanoic acid; C16:0, hexadecanoic acid; C17:0, heptadecanoic acid; C18:0, octadecanoic acid. Bar charts are plotted using mean ± SEM value, and statistical significance between both groups is defined as *p < 0.05; **p < 0.01; ***p < 0.005

The experimental procedure for establishing NMS model was portrayed in Fig. 1a. Compared with controls, increased stool frequency and shortened gut transit time were observed in adult rats subjected to NMS, suggesting enhanced GI motility (Fig. 2a, b). There was no difference in body weight between the groups (Additional file 1: Figure S1C), but fecal samples of NMS rats showed higher water contents and lower pH (Additional file 1: Figure S1A and B), which can be interpreted as abnormal fecal characteristics. The fecal volatile organic compounds (VOCs) were profiled by non-targeted metabolomic analysis by gas chromatography coupled with mass spectrum (GC-MS). Partial least squares-discriminant analysis (PLS-DA) showed pronounced differences in the profiles of fecal VOCs between the two groups (Fig. 2c). A total of 21 changed metabolites in NMS rats was identified referred by the variable importance in the projection (VIP) > 1 and *p* < 0.05 (Additional file 1: Table S1). Of these metabolites found, 11 were acidic substances closely associated with gut microbial fermentation and energy metabolism. Particularly, the six saturated fatty acids (SFAs) of the two groups differed dramatically, with increase of heptadecanoic acid (C17:0), tetradecanoic acid (C14:0), pentadecanoic acid (C15:0), and acetic acid (C2:0) while decrease of butyric acid (C4:0) and valeric acid (C5:0) in the NMS rats (Fig. 2d). These alterations suggest disturbance of the luminal SFA composition in NMS rats. Further, targeted SFA analysis found that the level of total SFAs was remarkably increased in the colon lumen of NMS rats (Fig. 2e). The cumulative levels of saturated short-chain fatty acids (from C2:0 to C5:0) and medium-chain fatty acids (from C6:0 to C12:0) were unchanged (Fig. 2f), but the level of total and individual SLCFAs (from C14:0 to C18:0) showed significant over-representation (Fig. 2f, g). These results suggest that an excess of intracolonic SLCFAs is associated with enhanced GI motility in NMS rats.

### SLCFAs stimulate rat colonic contraction and defecation

**Fig. 3:**
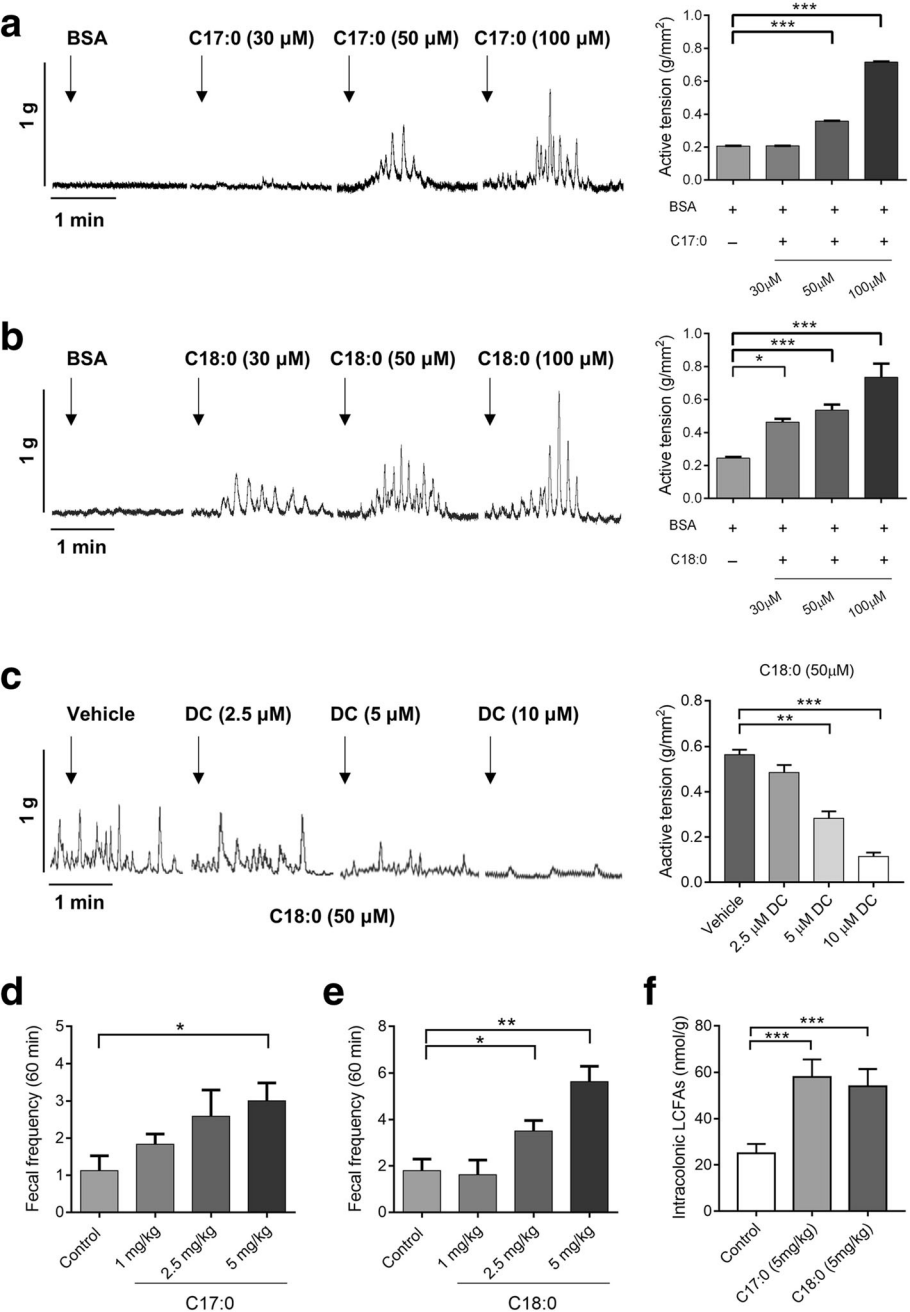
C17:0 and C18:0 dose-dependently stimulate colonic motility ex vivo and in vivo, and such SLCFA-induced muscle contraction can be attenuated by TLR4 inhibitor C34. **a, b** The effects of C17:0 and C18:0 on the contraction of circular muscles of isolated colon segments through an organ bath system. The amplitude of contractions was expressed as force/area (g/mm^2^). **c** The effects of selective GPR40 antagonist DC260126 (DC) on C18:0-induced colonic muscle contraction. **d, e** The effects of C17:0 and C18:0 on the accumulation of fecal pellet output within 60 min in normal rats (n = 8/group). **f** The accumulated amounts of SLCFAs per gram of colonic contents in SLCFA-treated and vehicle rats. Statistical differences among individual groups were evaluated using One-way ANOVA, and significance is defined as *p < 0.05; **p < 0.01; ***p < 0.005

Lipid perfusion has been shown to enhance colon motility in normal subjects and patients with IBS-D [26]; however, the effects of SLCFAs on colonic motility have not been reported. To determine these effects, we studied C17:0 and C18:0, the most abundant even- and odd-numbered carbon SLCFAs in the colon lumen of NMS rats, using an organ bath system as described in our previous study [27]. Compared with baseline (fatty acid-free BSA), C17:0 (50 and 100 μM) and C18:0 (30, 50, and 100 μM) dose-dependently enhanced contraction amplitudes of colonic circular muscles (Fig. 3a, b). Acetylcholine treatment was used as the positive control (Additional file 1: Figure S2A). C2:0, another saturated FA found to be increased level in fecal VOCs of NMS rats, was tested as a negative control (Additional file 1: Figure S2B). It is well known that GPR40 and GPR120, expressed in the endocrine cells of the colon, can be activated by free LCFAs [28–30]. To test which free long-chain fatty acid receptors are involved in such SLCFA-induced muscle contraction, isolated colonic segments were separately treated with different doses of DC260126 (GPR40 antagonist) and AH7614 (GPR120 antagonist) prior to introduction of C18:0. We found that DC260126 in doses of 5 and 10 μM can effectively suppress C18-induced colonic contraction, but AH7614 showed no effects (Fig. 3c and Additional file 1: Figure S2B). Furthermore, this effect of SLCFAs on colonic motility was confirmed in normal rats in vivo through orally administrating SLCFAs at dosages of 1, 2.5, and 5 mg/kg. Both C17:0 (5 mg/kg) and C18:0 (2.5 and 5 mg/kg) notably increased rat defecation frequency (Fig. 3d, e). The cumulative amount of intracolonic SLCFAs was significantly increased in rats given 5 mg/kg of C17 or C18 (Fig. 3f), and this amount is close to the total SLCFA level in the colonic contents from NMS rats. These findings revealed that SLCFAs can accelerate rat colonic motility, and such action is possibly involved in GPR40 signaling.

### Changed fecal microbiome is associated with SLCFA generation in NMS rats

Generally, intraluminal SLCFAs are mainly derived from diets or synthesized by host and gut microbiota. A previous study screened six genes (*Fasn*, *Scd1*, *Srebp1*, *CD36*, *Fabp2*, and *Cav1*) related to lipogenesis and lipolysis based on RT-PCR array and found no difference in their levels between adult NMS and control rats [31]. These evidences indicate that NMS rats have normal levels of host fatty acid synthesis, digestion, and absorption. Another piece of evidence has revealed that odd-numbered carbon SLCFA C15:0 and C17:0 can be produced by bacteria only [32]. Hence, we hypothesized that increase of SLCFAs is associated with the gut microbiota.

**Fig. 4:**
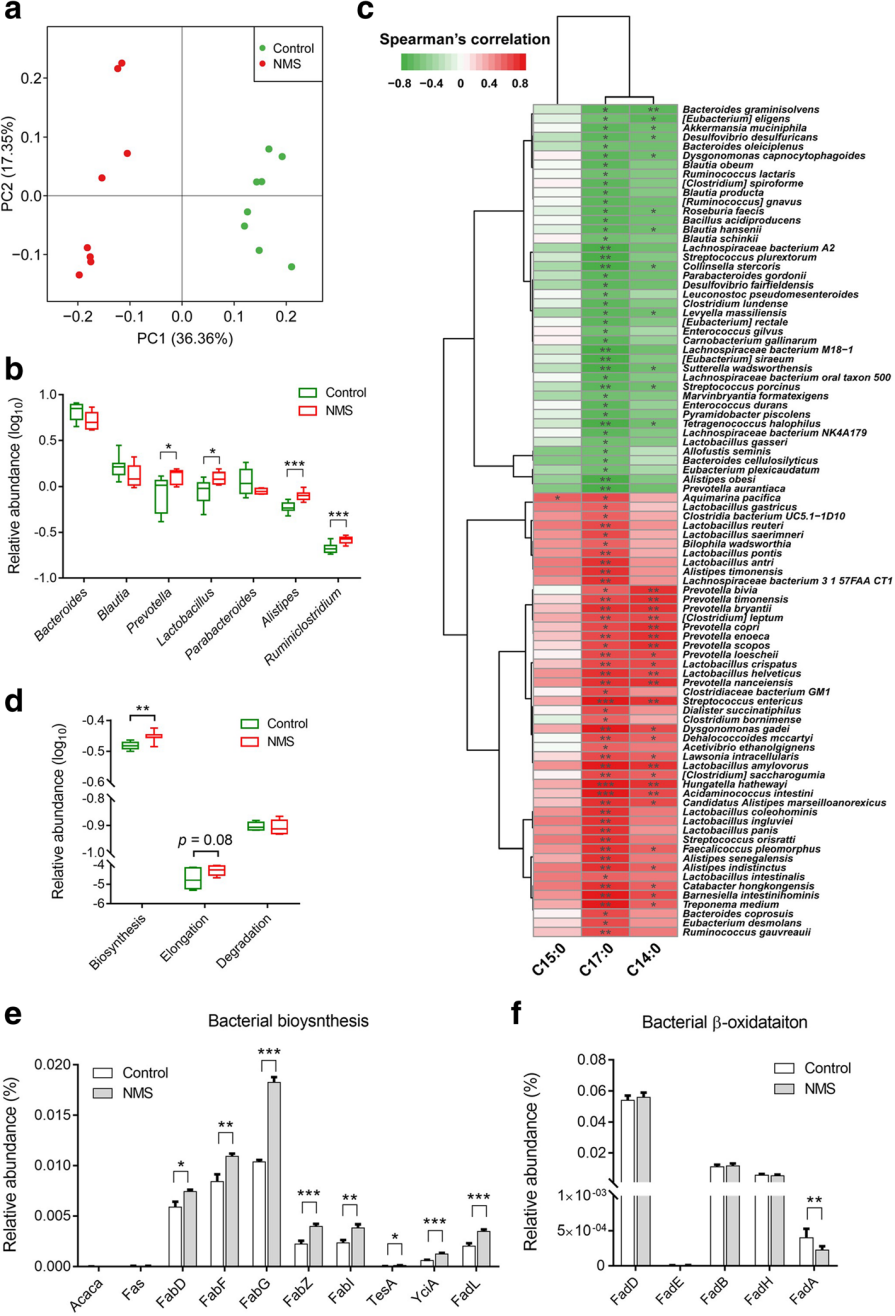
Alteration of fecal microbiome is closely associated with SLCFA generation in NMS rats (*n* = 8/group). **a** Principal coordinate analysis (PCoA) of microbial beta-diversity between both groups. The first two principal coordinates (PCs) respectively explain 36.36% and 17.35% of variation in the dataset. **b** The relative abundances of dominant genera in fecal microbiota. **c** The spearman’s correlation between changed fecal species and fecal C15:0, C17:0, C14:0. Only species correlated with at least one SLCFA with adjusted *p* < 0.05 are shown in the heatmap. **d** The relative abundances of fatty acid biosynthesis, elongation and degradation based on KO module analysis. **e** The relative abundances of genes encoding enzymes for SLCFA synthesis. **f** The relative abundances of genes encoding enzymes for β-oxidation. Differential abundance of taxa and KOs were evaluated by two-tailed Wilcoxon rank-sum test, and significance is defined as *p < 0.05; **p < 0.01; ***p < 0.005. Abbreviation: Acaca, acetyl-CoA carboxylase; Fas, fatty acid synthase (bacteria type); FabD, Malonyl CoA-acyl carrier protein; FabF, 3-oxoacyl-[acyl-carrier-protein] synthase II; FabG, 3-oxoacyl-[acyl-carrier protein] reductase; FabZ, 3-hydroxyacyl-[acyl-carrier- protein] dehydratase; FabI, enoyl-[acyl-carrier protein] reductase I; TesA, acyl-CoA thioesterase I; YciA, acyl-CoA thioesterase; FadL, long-chain fatty acid transport protein; FadD, long-chain acyl-CoA synthetase; FadE, acyl-CoA dehydrogenase; FadB, 3-hydroxybutyryl-CoA dehydrogenase; FadH, 2,4-dienoyl- CoA reductase; FadA, acetyl-CoA acyltransferase

Gut dysbiosis in NMS rodents has been previously reported based on 16s ribosomal RNA sequencing [24, 25]. However, such approach cannot be used to obtain information related to metabolic function. To investigate whether gut dysbiosis results in abnormal SLCFAs, and what the key manipulators are, we performed metagenomic sequencing analysis of fecal samples collected on postnatal day (PD) 56 from NMS and control rats (*n* = 8/group). In total, 83.01% of the high-quality sequencing reads (6.6 GB per sample on average) were used to generate 2.7 million contigs without ambiguous bases (minimum length of 500 bp), which allowed on average 74.75% of the reads in each sample to be mapped (Additional file 2: Table S2). Metagenomic results showed that microbial richness and α-diversity was reduced in NMS rats at gene level (Additional file 1: Figure S3A). Microbial β-diversity was distinct between both groups based on either principal coordinate analysis (PCoA) analysis (Fig. 4a) or Bray-Curtis dissimilarity (Additional file 1: Figure S3B). Taxonomy of the microbes was profiled at phylum, genus, and species levels (Additional file 2: Tables S3–S5). At the phylum level, *Bacteroidetes*, *Firmicutes*, *Proteobacteria*, *Deferribacteres*, and *Actinobacteria* dominated the fecal microbial communities of both groups, but without statistical differences (Additional file 1: Figure S3C). Taxonomic profiles of both groups were compared at genus level (Additional file 1: Figure S3D) and revealed that relative abundances of *Bacteroides*, *Blautia*, and *Parabacteroides* were slightly decreased, while abundances of *Prevotella*, *Lactobacillus*, *Alistipes*, and *Ruminiclostridum* were significantly increased in fecal microbiota of NMS rats (Fig. 4b). At species level, 168 species were significantly altered, and a majority of them (90 species) were found significantly correlated with at least one of the fecal SLCFAs (Additional file 2: Table S6). More specifically, multiple species from the genera *Prevotella*, *Lactobacillus*, and *Alistipes* were positively correlated with fecal C17:0 or/and C14:0 (Spearman’s correlation, adjusted *p* < 0.05; Fig. 4c and Additional file 2: Table S6). Moreover, pathway analysis of gene and genome (KEGG) orthologs (KO) found that the levels of bacterial fatty acid biosynthesis and elongation were highly enriched in the NMS model group, whereas fatty acid degradation level appeared no difference in NMS rats in comparison to controls (Fig. 4d). Ten genes mapped in fatty acid synthesis were identified in both groups. Of these, five genes, encoding to synthetases and elongases (FabD, FabF, FabG, FabZ, and FabI) responsible for bacterial LCFA synthesis and elongation [33, 34], showed elevated relative abundances in the NMS group (Fig. 4e). Moreover, abundances of genes *TesA*, *YciA*, and *FadL*, encoding to thioesterase and long-chain fatty acid transporter that takes charge of termination of LCFA elongation and LCFA transport across bacterial cytoplasmic membranes [35, 36], were also elevated in the NMS group (Fig. 4e). Notably, the abundant elongating *Fab* genes that are indispensable for LCFA generation were expressed in the genera *Prevotella*, *Lactobacillus*, and *Alistipes* (Additional file 2: Table S7), suggesting that they participate SLCFA formation. In addition, five *Fad* genes encoding to oxidase enzymes were mapped in fatty acid β-oxidation, one important pathway for LCFA degradation. The level of *FadA* was significantly reduced in the NMS group while other genes showed no difference (Fig. 4f). Taken together, metagenomic results suggest hyperactive bacterial SLCFA synthesis in NMS rats, with contributions from the genera *Prevotella*, *Lactobacillus*, and *Alistipes*.

### Fecal microbiota from NMS donors enhances stool frequency and intracolonic SLCFAs in pseudo GF rats

**Fig. 5:**
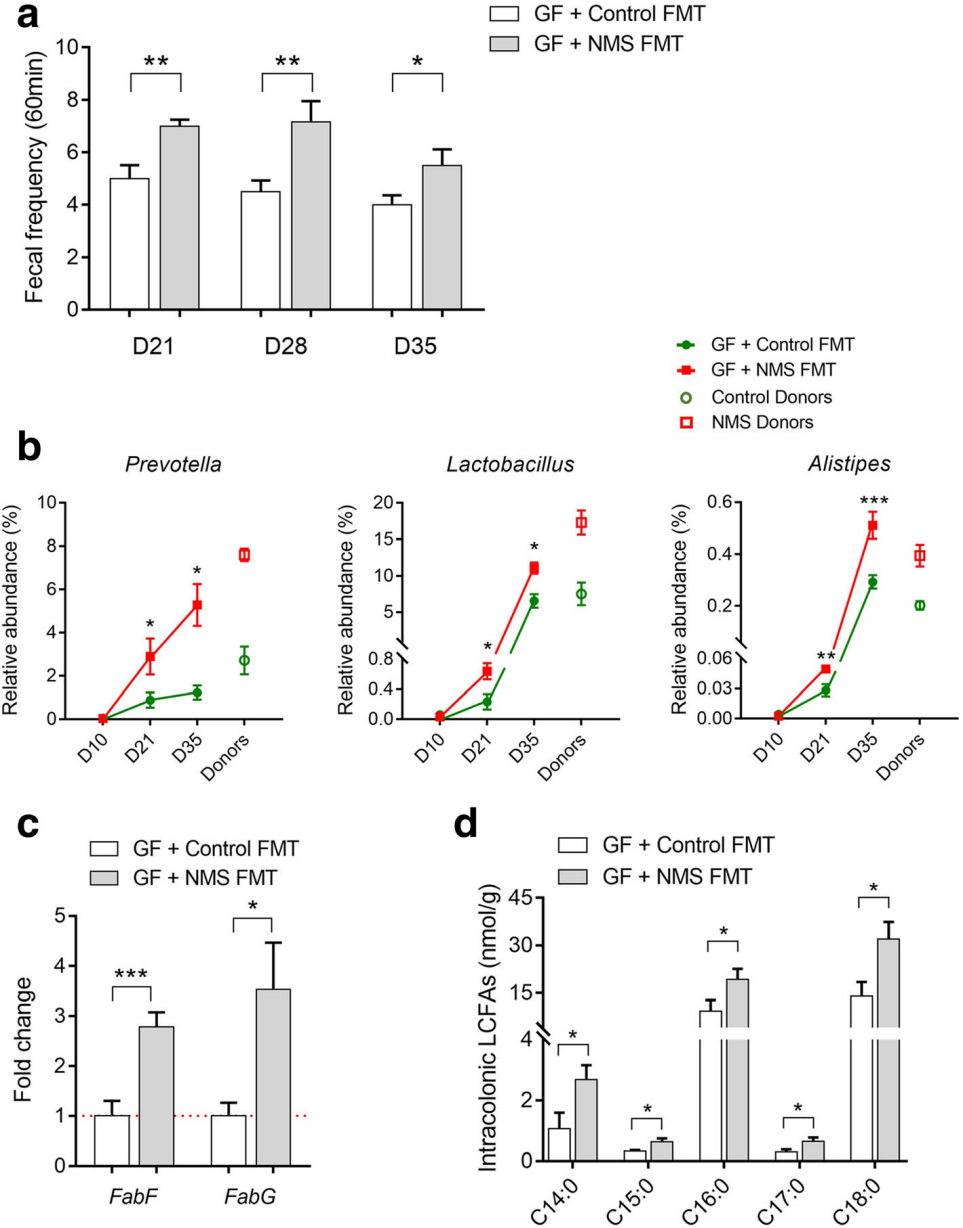
Fecal microbiota from NMS donors enhances defecation frequency and intracolonic bacterial SLCFAs production in pseudo germ-free (GF) rats (*n* = 6/group). **a** Accumulation of fecal pellets within 60 min in colonized GF rats 1 week (day 21), 2 weeks (day 28), and 3 weeks (day 35) after microbial transplant. **b** Relative abundances of fecal SLCFA-producing genera in donors and colonized GF rats on days 10, 21, and 35. **c** The relative levels of genes required for SLCFA synthesis in colonic contents of colonized GF rats. **d** The amounts of individual SLCFAs per gram of colonic contents. Bar charts are plotted using mean ± SEM value, and statistical significance between both colonized groups is defined as *p < 0.05; **p < 0.01; ***p < 0.005

To investigate whether gut microbiota of NMS rats promotes SLCFA production in the colon lumen and enhances bowel motility, fecal microbiota prepared from NMS or NH donors were transplanted to pseudo GF rats (*n* = 6/group). The experimental process is showed in Fig. 1b. The pseudo GF model was induced by providing rats with water spiked with antibiotic cocktail (ABX), according to a published method [37]. During GF model establishment, rats from different cages consumed similar volumes of ABX water (Additional file 1: Figure S4A), indicating there would be no difference in bactericidal action for GF modeling. Fecal microbiota extracted from donors were prepared as PBS suspensions, and orally administrated to pseudo GF rats for 5 consecutive days. One week after fecal microbial transplant (FMT), accumulation of fecal output within 60 min was found to be significantly increased in GF rats colonized with microbiota of NMS donors (NMS FMT) relative to controls that has been colonized with microbiota of control rats (Control FMT); the change was maintained over the following 2 weeks (Fig. 5a). In addition, the baseline of defecation number was higher in GF recipients than conventional rats, which possibly relates to watery or shapeless stools presumably caused by continuous infusion of ABX water. A similar fecal phenotype has been reported in other antibiotic-induced GF studies [38, 39].

Microbial DNA extracts from fecal samples of GF rats on day 0 (baseline), day 10 (after ABX treatment), day 21 (1 week after transplant), and day 35 (3 weeks after transplant) were subjected to 16s ribosomal RNA sequencing analysis to determine the dynamic change of fecal enterotypes (Additional file 2: Table S8). Compared with the baseline, dramatic loss of microbial richness and DNA integrity observed in ABX-treated rats indicates that the antibiotic-induced pseudo GF model was well-developed (Additional file 1: Figure S4A-C). The ecological community of feces collected from colonized GF rats was then compared with that of the donors from diversity and taxonomic perspectives. PCoA analysis showed a certain similarity of bacterial β-diversities between recipients and donors (Additional file 1: Figure S4D). Specifically, changes of 49 genera from NMS donors, accounting for 50% of identified bacteria, also appeared in GF rats with NMS FMT (Additional file 2: Table S9).

Although the bacterial α-diversity from both groups of recipients was similar (Additional file 1: Figure S4B), both β-diversity and bacterial composition in GF rat with NMS FMT were certainly distinct from those that had received control FMT (Additional file 1: Figure S4D and E). Particularly, relative abundances of *Prevotella*, *Lactobacillus*, and *Alistipes* were significantly increased after FMT, and such bacterial profile of GF recipients on day 35 was closer to that of donors (Fig. 5b). Quantitative polymerase chain reaction (qPCR) analysis found that relative levels of two indispensable LCFA-synthesized genes *FabF* and *FabG* were increased in the colon lumens of GF rats receiving fecal microbiota of NMS donors on day 35 (Fig. 5c). Consistently, the levels of individual SLCFAs (from C14:0 to C18:0) were obviously raised in the colon lumen (Fig. 5d). These results indicate that featured enterotype of NMS rats could result in elevation of stool frequency and SLCFA generation.

### Microbial modulation effectively reduces GI motility and intracolonic SLCFA production in NMS rats treated with neomycin

**Fig. 6:**
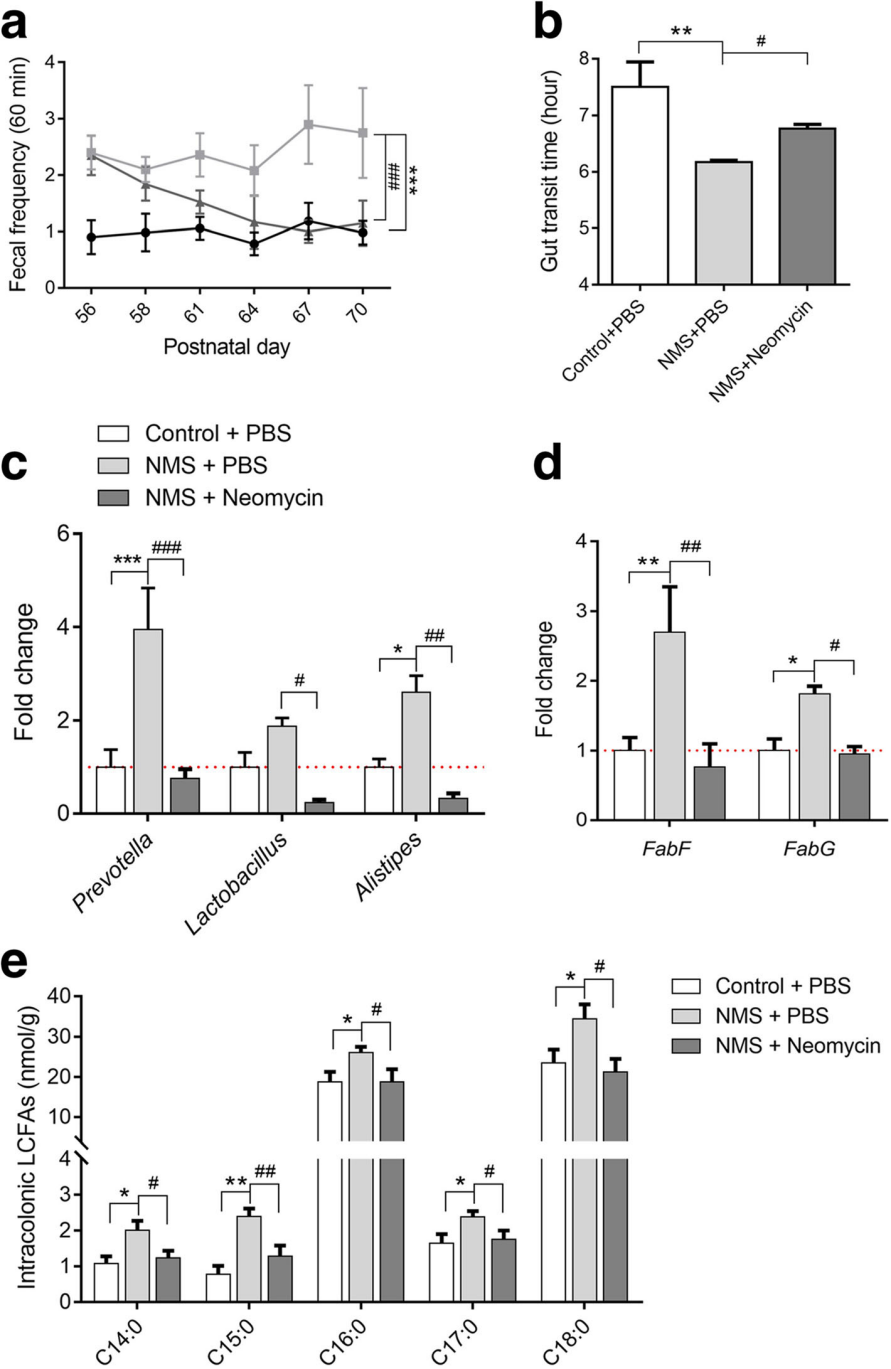
Modulation of gut microbiota can effectively attenuate bowel motility and intracolonic SLCFAs production in NMS rats based on 14 consecutive days of neomycin treatment (150 mg/kg). **a** Accumulation of fecal pellets within 60 min before and after neomycin treatment. **b** The gut transit time in rats with or without intervention. **c** Relative levels of SLCFA-producing genera normalized with 16s rRNA level. **d** Relative levels of SLCFA-synthetic genes among three groups. **e** The amounts of individual SLCFAs per gram colonic contents in rats from three groups. Values for line/bar charts are expressed as mean ± SEM, for box plots are presented as interquartile range. Statistics among three groups was calculated by One-way ANOVA. Significance compared with control group is defined as *p < 0.05; **p < 0.01; ***p < 0.005, compared with NMS group is described as #*p* < 0.05; ##*p* < 0.01; ###*p* < 0.005

To further confirm the relationship between SLCFA-producing bacteria and bowel motility, the antibiotic drug neomycin, previously reported as efficient in the modulation of gut dysbiosis and improvement of bowel symptoms in patients with IBS [40, 41], was chosen to orally administrate to NMS rats (150 mg/kg), twice daily, from postnatal days (PD) 56 to 70 (Fig. 1a). Control groups were treated with PBS. Stool frequency was monitored every 3 days during drug intervention. As shown in Fig. 6a, model rats with oral gavage of PBS presented greater stool pellet output within 60 min. Enhanced defecation number was effectively attenuated and ultimately returned to normal after 14 days of neomycin treatment. The gut transit time became prolonged in neomycin-treated NMS rats, but was shorter than control rats (Fig. 6b). Genus-specific PCR analysis found that levels of *Prevotella*, *Lactobacillus*, and *Alistipes* were downregulated in the colon lumens of the neomycin-treated group (Fig. 6c). Also, the levels of SLCFA synthetic genes *FabF* and *FabG* were reduced (Fig. 6d). In line with changes of SLCFA-producing bacteria and genes, intracolonic levels of individual SLCFAs were notably attenuated after intervention (Fig. 6e).

## Discussion

In this study, we observed an altered gut microbiota in NMS rats characterized by increased capacity for generation of SLCFAs, which were proved to enhance rat colonic contraction ex vivo and stool frequency in vivo. Further, with microbiota intervention studies including FMT and antibiotic treatment, we found that SLCFA-producing bacteria contribute to the acceleration of colonic motility in rats.

SLCFAs have been reported to regulate GI motility in humans, most of studies concerned their effects on the upper GI tract. A study of 10 healthy men showed that upper intestinal infusion of oils enriched with C18:0 significantly reduced gastric motility [42]. The similar inhibitory impact of SLCFAs on the upper gut motility has also been reported in patients with FGIDs [43]. LCFA-induced slower upper GI motility influences food intake and energy metabolism, demonstrating the importance of SLCFAs in maintenance of body weight as well as in obesity progression [44]. However, we found no difference in the body weight and luminal SLCFAs in the small intestine between NMS rats and controls. A previous study found that increase of defecation was not accompanied by a concomitant change in food consumption in NMS model [45]. Thus, the upper GI motility is possibly not affected by SLCFAs in the NMS model.

The effects of LCFAs on the lower GI tract, i.e., the colon, is another story. Some studies have reported that lipids can increase colon motility in normal subjects and patients with IBS-D [26, 43]. Infusion of unsaturated LCFAs have been shown to accelerate colon transit in human [46, 47]. But the effects of SLCFAs specifically on the colon motor function have not been reported. This study is the first to provide direct evidence for the promotional action of SLCFAs on colon motility. Compared with SLCFAs, short-chain fatty acids (C3:0 and C4:0) show low potency for stimulating the colonic motor [48]. Also, we found that C2:0, present increased level in fecal VOCs of NMS rats, cannot induce muscle contraction of the isolated colon. This suggests that LCFAs have distinct and specific effects on the colon. Furthermore, elevated muscle contraction induced by C18:0 can be significantly inhibited by a GRP40 antagonist but not a GRP120 antagonist, indicating that GPR40 is specifically involved in the SLCFAs-stimulated colon motility. Briscoe et al. have reported that fatty acids with carbon chain lengths greater than six are able to activate GPR40, thereby giving rise to elevation of calcium secretion and release [49]. Such GPR40-dependent Ca^2+^ rise can be blocked by inhibition of L-type calcium channels or opening of the K(ATP) channel [50]. These evidences suggest that LCFAs stimulate colon motility possibly through upregulation of GPR40-dependent Ca^2+^ influx. We intend to explore this possibility in further study.

Generally, luminal SLCFAs are derived from either lipolysis of dietary fats or host/bacterial fatty acid synthesis [43, 51, 52]. A previous study reports normal expression of enzymes for host lipolysis, transport and lipogenesis at the mRNA level in NMS model rats [31]. Moreover, SLCFAs with odd-numbered carbon atoms, such as C15:0 and C17:0, can be produced by bacteria only [32]. If this is true then, elevation of individual SLCFAs from C14 to C18 in the colon lumens of NMS model should be the results of gut bacterial actions. In our study, the combination of metabolomic and metagenomic results points to a significant correlation between changes of dominant bacteria and levels of excretive SLCFAs. KO analysis of fecal metagenome showed obvious elevation in the level of fatty acid synthesis and elongation. These findings also support the hypothesis that gut bacteria engage in formation of intracolonic SLCFAs.

Bacterial fatty acid synthesis is essential for supplying the hydrophobic components of the membrane lipids and for providing components of storage lipids [53]. The synthetic process, mainly including initiation and elongation, is executed by a cluster of Fab enzymes that are strongly conserved in bacteria [33, 34, 54]. Of these, FabF and FabG are indispensable for generation of LCFA with carbon atoms 14 to 18 [55, 56]. In a previous study of alcoholic liver disease, the gene expression level of FabF and FabG has been determined to reflect the level of bacterial LCFA synthesis in humans and mice [57]. We observed that genomic levels of *FabF* and *FabG* were highly enriched in fecal microbiota of NMS rats. Such increased levels of *FabF* and *FabG* also appeared in GF rats receiving fecal microbiota from NMS donors, but were attenuated upon modulation of NMS-associated gut dysbiosis. These observations indicate that a higher level of bacterial SLCFA synthesis is characteristic of the NMS-associated enterotype.

The genera *Prevotella*, *Lactobacillus* and *Alistipes* expressing essential *Fab* genes showed elevated abundances in NMS rats and GF rats receiving fecal microbiota of NMS donors, and were significantly reduced in NMS rats after neomycin treatment. Probably, these three genera are important manipulators for intracolonic SLCFA disruption. Interestingly, these SLCFA-producing bacteria have been linked with host GI motility both in clinical and laboratory studies. For example, abundances of *Lactobacillus*, *Prevotella* and *Alistipes spp.* are significantly decreased in patients with constipation [10–12]. Conversely, increase of the genus *Lactobacillus* has been reported in patients of IBS-D [58]. Higher abundances of *Prevotella spp.* are found in c*hildren with chronic diarrhea* [59]. The genera *Prevotella*, *Lactobacillus* or *Alistipes* showed increased abundance in chronic stress-induced rodents characterized by GI dysmotility [24, 25]. *Lactobacillus* strains accelerate intestinal transit in GF rats and enhance ileal contraction in guinea-pig [60, 61]. These lines of evidence consistently support a linkage between SLCFA-producing bacteria and host colonic motility; however, further studies are needed to identify the precise roles of specific species or strains in modulating host colonic motility.

By profiling fecal VOCs, we noticed that acidic substances, including SLCFAs and fermented products (e.g., lactate and acetate) were significantly elevated in NMS group, corresponding to the raised acidity in stools. Meanwhile, increase of fecal TCA intermediates citrate and succinate in NMS rats suggest a higher metabolic level of citrate cycle. It is supported by fecal metagenomic results that a significant enrichment of citrate cycle module in NMS group relative controls (different genes mapping in TCA cycle: 398 genes for control and 486 genes for NMS group, adjust *p* < 0.05). Of them, succinate can stimulate water secretion from intestinal segments [62], indicating that increased fecal consistency of NMS rats can be attributed to altered microbial metabolites. In contrast, fecal butyrate and valerate showed reduced levels in the NMS group. Previous study has found that butyrate is beneficial to maintenance of the intestinal epithelial barrier through facilitating tight junction assembly [63]. Soderholm et al. revealed that NMS enhances vulnerability of the colonic mucosal barrier to stress [64]. Probably, deficiency of bacterial butyrate production is one cause of the weak colonic barrier of the NMS model. This evidence indicates the importance of bacterial metabolites in controlling both stool characteristics and colonic barrier function.

## Conclusion

This study determined that NMS rats have excessive levels of intracolonic SLCFAs, and these SLCFAs are specifically related to accelerated colonic movement and increased fecal output. Such elevated SLCFAs is contributed by hyperactive synthetic action of gut bacteria, specifically by the genera *Prevotella*, *Lactobacillus* and *Alistipes*. Fecal microbial transplantation and antibiotic modulation revealed a causal relationship between SLCFA-producing bacteria and host colonic motility. These findings clarify the stimulatory effects of SLCFAs on the colon motility and provide novel insight in gut microbiota-driven GI dysmotility, which may lead to a therapeutic intervention targeting specific gut microbiota for treating GI motility disorders.

## Methods

### Animals

All Sprague-Dawley (SD) rats used for this study were obtained from the Laboratory Animal Services Centre of The Chinese University of Hong Kong. Rats were maintained on a 12-h light/dark cycle with free access to food and water under specific pathogen-free (SPF) condition. Materials including cages, diets, water, and litters were sterilized, and stool frequency was tested in a clean hood with a UV lamp for pseudo GF rats. All animal experiments have followed the Animals Ordinance, Department of Health, Hong Kong SAR, China.

### Neonatal maternal separation modeling and colonic motility assessment

Referring to our previous study [65], the procedure of the NMS model was performed as shown in the timeline (Fig. 1a). Briefly, four pregnant SD rats were housed individually in cages. Randomly, newborn pups from two mothers were assigned to NMS group and pups from another two dams were assigned to non-handled control group. NMS pups were separated from their dams during the period of postnatal days (PD) 2 to 14 for 3 h daily whereas control pups remained with their mothers constantly. All pups were weaned on PD 22, and only male pups (*n* = 8/group) with similar body weight was used for further experiments. GI motility was individually evaluated in rats 8 weeks later. Fecal pellets were collected to measure pH level and water contents, and were used for analyses of metagenomics and metabolomics. The detail methods for assessment of motor function and stool characteristics were described in Additional file 1. The luminal contents of ileum (10 cm proximal to the cecum), cecum, and proximal colon (5 cm distal to the cecum) were collected after CO_2_ anesthesia, and were immediately stored at − 80 °C. Furthermore, to investigate the relationship between SLCFA-produced bacteria and the colonic motor in NMS rats, the drug neomycin (150 mg/kg) was twice daily administrated to adult NMS rats for 14 consecutive days (from PD 56 to 70). Meanwhile, other NMS or control rats were orally gavaged with PBS.

### Pseudo germ-free modeling and fecal microbial transfer

The experimental procedure is shown in Fig. 1b. The antibiotic cocktail (ABX), containing ampicillin (1 g/L), neomycin (1 g/L), and metronidazole (0.5 g/L) were prepared in drinking water as previously described [37]. The ABX-contained water was ad libitum supplied for 12 normal SD rats (*n* = 6/group) to establish the pseudo GF model. No difference in the record of daily and total water consumption (Additional file 1: Fig. S4A) indicates that rats in different cages ingested similar volumes of ABX. To ensure elimination of the gut microbiome, fecal pellets were frequently collected during experiment for real-time monitoring of dynamic changes of gut microbiota. The very low levels of fecal total DNA quality and bacterial diversity (Additional file 1: Fig. S4B and C) indicate that the pseudo GF model was successfully established after 10 days of ABX-water intervention. Furthermore, donors’ feces from either NMS or control group (*n* = 8/group) were individually pooled and completely homogenized in pre-reduced PBS at 1 ml per pellet referring to a published method [20]. One milliliter of the settled suspension was daily administered to pseudo GF rats for five consecutive days. The stool frequency was weekly determined in colonized GF rats. Fecal samples were collected on Day 0, 10, 21 and 35 for further microbial analysis, and colonic contents were collected on Day 35 for assessment of SLCFA producing genes and SLCFA amounts.

### Preparation of saturated long-chain fatty acids for colonic motility assessments

To evaluate the effects of SLCFAs on host colonic motility, dominant odd- and even-numbered carbon SLCFAs C17:0 and C18:0 were chosen to individually test their influences on the muscle contraction of rat isolated colon segments ex vivo and rat stool frequency in vivo. C17:0 and C18:0 (Cat#: 506-12-7 and 57-11-4, Sigma-Aldrich, St. Louis, MO USA) were solubilized with fatty acid free-bovine serum albumin (BSA, Cat#: 9048-46-8; Sigma-Aldrich, USA) to generate a series of concentrations (10, 30, 50 and 100μM) for organ bath system-based colonic contraction test ex vivo [66]. The concentration range were calculated in accordance with SLCFA levels found in the colon contents of NMS rats with 1.1 g/cm3 of density of the colonic contents reported in a previous study [67]. To test which long-chain fatty acid receptors mediate SLCFA-stimulated colonic contraction, selective GPR40 antagonist DC260126 (DC, Cat#: 346692-04-4, Tocris Bioscience, UK) and GPR120 antagonist AH7614 (AH, Cat#: 6326-06-3, Tocris Bioscience, UK) was prepared at working dosages of 2.5, 5, and 10μM according to previous studies [68, 69]. Acetylcholine (Ach, Cat#: 60–31-1, Sigma, St. Louis, MO USA) and C2:0 (Cat#: 127-09-3, Sigma, St. Louis, MO USA) were applied as positive and negative control, respectively. The experimental procedure was described in Additional file 1. Furthermore, both SLCFAs were prepared as different dosages (1, 2.5 and 5 mg/kg, dissolved in 5% ethanol) for in vivo measurement of stool frequency in normal SD rats (*n* = 8/group). Each rat orally treated with either 1 mL of SLCFAs or vehicle solution was located in an individual cage for defecation frequency assessment. Fecal pellets were collected for determination of intracolonic SLCFA levels.

### Total DNA isolation and metagenomic sequencing

Total bacterial DNA was isolated and purified from fecal pellets (precisely weighted 200 mg) using a stool DNA Isolation Kit (Qiagen, Valencia, CA). All samples were sequenced based on the Illumina Hiseq 4000 platform (paired-end; insert size, 350 bp; read length, 150 bp). After removal of adaptor and low-quality reads, the remaining reads were filtered to eliminate the host DNA genome based on the genome reference of *Rattus norvegicus* by SOAPalign v2.21 [70]. Finally, 105.81 Gb high-quality pair-end reads for the 16 rats samples (*n* = 8/group) was acquired with an average of 6.61 Gb per sample in groups of NMS and control (Additional file 2: Table S2).

### Construction of the gene catalog

The reads were assembled into contigs for all samples using the assembly software SOAPdenovo v2.0455. Average 75.67% of the total reads were used to generate 1.6 million contigs without ambiguous bases (minimum length of 500 bp). ORFs were predicted from the assembled contigs using the MetaGeneMark v3.26 programme [71]. The 6,116,823 ORFs longer than 100 bp covered 89.67% of the total length of the contigs and about half (50.51%) of the ORFs appeared complete. All ORFs were clustered by CD-hit v4.6.4 to construct a non-redundant gene catalog using a stringent criterion of 95% identity at the nucleotide level over 90% of the length of the shorter ORFs [72]. The final non-redundant gene set contained 1,462,418 ORFs with an average length of 728 bp.

### Diversity analysis, taxonomic assignment and functional characterization

The α-diversity (within-sample diversity) and β-diversity (between-sample diversity) were estimated by the Shannon index and Bray-Curtis dissimilarity metric, respectively [73]. For taxonomic assignment, all predicted genes were blasted against the reference microbial genomes from NCBI (including 4258 microbial genomes) by using BLAST (v 2.2.26, default parameter) with at least 80% overlap of query. Taxonomic identification was performed as 65% identity for phylum, 85% identity for genus, 95% identity for species [74, 75]. The taxonomic abundance was calculated based on gene abundance, and protein sequences of the predicted genes were searched using National Center for Biotechnology Information BLASTP against the KEGG gene database (v79). Each protein was assigned to the KEGG group by the highest scoring annotated hits. Significance in the relative abundance of genes, KOs, phylum, genera, and species between both groups were compared by two-tailed Wilcoxon rank-sum test (Additional file 2: Tables S3-S7). The relationship between the abundance of each species and SLCFAs contents was assessed by the Spearman’s correlation [76]. Enrichment in NMS or control group was then determined according to the higher rank-sum.

### Determination of bacterial DNA quality and 16s ribosomal RNA amplicon sequencing

DNA extracts was prepared from feces (precisely weighted 200 mg) of donors and colonized GF rats. The DNA yield and quality were determined spectrophotometrically by the NanoDrop™ ND-2000 (Thermo Fisher Scientific Ltd., Waltham, MA, USA). The DNA integrity was determined through 1% agarose gel (*w*/*v*). Furthermore, the extracted DNA (30 ng) was amplified with universal primers (515F and 806R) to obtain the V4 regions of the 16S rRNA gene. The PCR products were purified and sequenced on the Illumina Hiseq 2500 platform. After raw data filtering and merging [77, 78], the numbers of tag and operational taxonomic unit (OTU) were generated from 50 samples (Additional file 2: Table S8). Bacterial diversity and taxonomy was obtained from OTU table by using QIIME software package [79].

### PCR analysis of genus-specific bacterial 16s rDNA and SLCFA synthetic genes

Genus-specific primers for *Prevotella*, *Lactobacillus* and *Alistipes* were obtained from published studies [32, 80]. Bacterial DNA extracts (50 ng) of colonic contents were amplified with pairs of primers and Power SYBR GREEN Master Mix (Applied Biosystems, Foster city, CA, USA) based on an ABI StepOne Plus Sequence Detection System (Applied Biosystems, Foster city, CA, USA). The cycling conditions were as follows: 95 °C for 10 min, followed by 40 cycles of 95 °C for 30 s, 52 °C for 30 s, and 72 °C for 1 min. Moreover, such SYBR GREEN-based PCR detection also applied to testify expression of LCFA synthetic *FabF* and *FabG* genes from colonic contents. The primers for *FabF* and *FabG* were designed as previously described [57]. The expression of each genus and gene was normalized to the level of 16S rRNA.

### Metabolite extraction and SLCFA quantification

Fecal samples or luminal contents (100 mg) were completely homogenized with five-fold volume of ice-cold distilled water. After high-speed centrifugation (13,000 rpm for 15 min at 4 °C), water extractions were transferred to a new 2-mL tube. Subsequently, a five-fold volume (500 μL) of methanol was added into the pellet sample. The mixture was completely homogenized and centrifuged again. Methanol extractions were combined with the previous water extractions. Strongly vortex and centrifugation again, the resulting supernatants were obtained for derivatization processing. Meanwhile, a quality control (QC) sample pooling all rat samples was prepared using the same protocol. For quantification of luminal SFAs in the small and large intestines, external calibration solution of SFA standards from C2:0 to C18:0 were prepared at a series of concentrations (from 1 to 20,000 ng/ml). Isotope labeled C_13_-myristic acid was used as internal standards. Absolute quantities of individual SFAs were normalized to the sample weight.

### GC/MS-based metabolomic analysis and data processing

Fecal metabolites were derivatized by BSTFA with 1% TMCS based on a previously method [81]. A gas chromatography coupled with a mass spectrum (GCMS-QP2010 systems, Shimaduzu Co., Tokyo, Japan) was applied for fecal metabolome analysis. Fecal derivatives were separated by a DB-5 MS fused-silica capillary column (30 m × 250 μm i.d.; Agilent J&W Scientific, Folsom, CA), chemically bonded with a 5% phenyl-95% methylpolysiloxane cross-linked stationary phase (0.25 μm film thickness). The detail sample preparation and analytical parameters of instrumental conditions were showed in Additional file 1. The metabolic signals were deconvoluted, aligned and normalized to final data matrix through using R-scipt with xcms package (R version 3.4.2). The processed matrix was introduced into the software SIMCA-P (Version 11.0, Umetrics, Umea, Sweden) for principal component analysis (PCA) and partial least squares-discriminant analysis (PLS-DA). Metabolic features were selected by combination of the variable importance in the projection (VIP) threshold (VIP > 1.0) and the Student’s *t*-test (*p* < 0.05). Metabolites were identified by the national institute of standards and technology (NIST) library (over 95% matched similarity).

### Statistical analysis

For sequencing data, statistical analyses were performed in R (v3.4.10) software; differential abundance of genes, taxonomies, and KOs were evaluated by two-tailed Wilcoxon rank-sum test. For determination of metabolites, genes, and colonic motor in animal studies, Mann-Whitney test was used for statistics between two groups, and one-way ANOVA was used for comparison among more than two groups. These statistical analyses were performed in GraphPad Prism 6 (GraphPad software lnc., CA, USA). Statistical significance is defined as *p* < 0.05.

## Additional files

Additional file 1 **Figure S1.** Fecal characteristics and body weight in NMS rats and controls. **Figure S2.** Muscle amplitudes of rat isolated colonic segments with different treatment. **Figure S3.** Phylogenetic profiles of fecal microbiomes of NMS and control rats. **Figure S4.** Fecal microbial community in pseudo GF rats at duration of FMT experiment. **Table S1.** Fecal metabolites with significant difference between NMS rats and controls. (DOCX 784 kb)

Additional file 2 **Table S2.** Data production, quality control, assembly result and gene prediction resulted from fecal metagenomic sequencing analysis. **Table S3.** Taxonomic profiles of fecal microbiota in NMS and control groups at phylum level. **Table S4.** Taxonomic profiles of fecal microbiota in NMS and control groups at genus level. **Table S5.** Taxonomic profiles of fecal microbiota in NMS and control groups at species level. **Table S6.** Correlation between species and saturated long-chain fatty acids determined by fecal metagenomic and metabolomic analyses. **Table S7.** Identified KOs involved in fatty acid synthesis and degradation. **Table S8.** Data production and quality control of fecal samples from 16s rRNA amplicon sequencing analysis. **Table S9.** Taxonomic changes at genus level between colonized GF rats and donors. (XLSX 73 kb)

## Abbreviations

ABX: Antibiotic cocktails
Acaca: Acetyl-CoA carboxylase
C14:0: Tetradecanoic acid
C15:0: Pentadecanoic acid
C16:0: Palmitic acid
C17:0: Heptadecanoic acid
C18:0: Octadecanoic acid
C2:0: Acetic acid
C4:0: Butyric acid
C5:0: Valeric acid
FabD: Malonyl CoA-acyl carrier protein
FabF: 3-Oxoacyl- [acyl-carrier-protein] synthase II
FabG: 3-Oxoacyl-[acyl-carrier protein] reductase
FabI: Enoyl-[acyl-carrier protein] reductase I
FabZ: 3-Hydroxyacyl-[acyl-carrier-protein] dehydratase
FadA: Acetyl-CoA acyltransferase
FadB: 3-Hydroxybutyryl-CoA dehydrogenase
FadD: Long-chain acyl-CoA synthetase
FadE: Acyl-CoA dehydrogenase
FadH: 2,4-Dienoyl-CoA reductase
FadL: Long-chain fatty acid transport protein
Fas: Fatty acid synthase (bacteria type)
NMS: Neonatal maternal separation
PCoA: Principal coordinate analysis
SLCFAs: Saturated long-chain fatty acids
TesA: Acyl-CoA thioesterase I
VIP: Variable importance in the projection
VOCs: Volatile organic compounds
YciA: Acyl-CoA 12 thioesterase
