# Supplementary material for: Saturated long-chain fatty acid-producing bacteria contribute to enhanced colonic motility in rats
Source: Microbiome. 2018 Jun 14;6:107. doi: 10.1186/s40168-018-0492-6 (PMC6003035; doi:10.1186/s40168-018-0492-6)
Supplement: Supplementary file 1 — Figure S1. Fecal characteristics and body weight in NMS rats and controls. Figure S2. Muscle amplitudes of rat isolated colonic segments with different treatment. Figure S3. Phylogenetic profiles of fecal microbiomes of NMS and control rats. Figure S4. Fecal microbial community in pseudo GF rats at duration of FMT experiment. Table S1. Fecal metabolites with significant difference between NMS rats and controls. (DOCX 784 kb) [file 40168_2018_492_MOESM1_ESM.docx]

**Additional file 1**

Saturated Long Chain Fatty Acid-Producing Bacteria Contribute to Enhanced Colonic Motility in Rats

Ling Zhao^1ǂ^, Yufen Huang^2ǂ^, Lin Lu^1ǂ^, Wei Yang^1^, Tao Huang^1^, Zesi Lin^3^, Chengyuan Lin^1,4^, Hiuyee Kwan^1^, Hoi Leong Xavier Wong^1^, Yang Chen^5^, Silong Sun^2^, Xuefeng Xie^2^, Xiaodong Fang^2,5^, Huanming Yang^6^, Jian Wang^6^, Lixin Zhu^7*^ and Zhaoxiang Bian^1*^

^ǂ^ Contributed equally to this work

^*^**Corresponding Author**

Zhao-xiang Bian, MD, PhD

E-mail: [bianzxiang@gmail.com](mailto:bianzxiang@gmail.com).

Lixin Zhu, PhD

E-mail: [lixinzhu@buffalo.edu](mailto:lixinzhu@buffalo.edu)

**This file includes:**

Supplementary experimental procedures

Fig. S1. Fecal characteristics and body weight in NMS rats and controls.

Fig. S2. Muscle amplitudes of rat isolated colonic segments with different treatment.

Fig. S3. Phylogenetic profiles of fecal microbiomes of NMS and control rats.

Fig. S4. Fecal microbial community in pseudo GF rats at duration of FMT experiment.

Table S1. Fecal metabolites with significant difference between NMS rats and controls.

**Supplementary experimental procedures**

*Measurement of fecal acidity and water contents*

For fecal acidity measurement, the fresh feces (100 mg) of all rats were immediately placed in 2 ml of cold distilled water. After homogenization, pH value was tested using a digital pH meter (Thermo Scientific, Orion, USA). Moreover, the wet and dry weights of each fecal pellet before and after drying in the oven (at 105°C for 24 h) were respectively recorded. Fecal water contents (%) was calculated by:

$Fecal water contents \left( \% \right)=$ $\frac{W_{wet}-W_{dry}}{W_{wet}}$ ×100

*Organ bath-based contraction of rat colon segments*

Ten normal male SD rats (250 ± 5g) were euthanized by asphyxiation with CO_2_. Fragments of the proximal colon were isolated and placed in Krebs solution (consisting of 119 mM NaCl, 4.5 mM KCl, 1.2 mM MgCl_2_, 25 mM NaHCO_3_, 1.2 mM KH_2_PO_4_, 2.5 mM CaCl_2_ and 11.1 mM glucose) at room temperature. An organ bath system bubbled with a mixture of 95% O_2_ plus 5% CO_2_ were maintained at 37 °C to assay the effects of saturated LCFAs on the colonic contraction. About 1.5 cm long piece of the tissue was horizontally placed in the organ bath. The colonic segments were equilibrated for 1 hour prior to the experiment with the washing step every 20 min with Krebs solution. The active tension of circular smooth muscle was recorded using the POWERLAB system and CHART5 software (AD instrument Ltd., Bella Vista, NSW, Australia). The circular amplitude of contraction was measured and expressed after treatment of BSA-solubilized C17:0 and C18:0 with different concentrations (10, 30, 50 and 100μM). Acetylcholine (ACh) with a concentration of 10 μM was used as positive control. C2:0 with same doses as SLCFAs (50 and100µM) were tested as negative control. Moreover, different doses (2.5, 5 and 10μM) of selective inhibitors DC260126 and AH7614, targeting on GPR40 and GPR120 respectively, were added into each channel thirty minutes prior to introducing 50μM of C18:0 for clarifying which long chain fatty acid receptor is involved in such SLCFA-stimulated colonic motility. The vehicle group was treated with 1% DMSO. The amplitude of muscle contraction was expressed as force/area (g/mm2).

*Evaluation of GI motility in vivo*

The GI motility was determined through evaluating gut transit time and defecation frequency. The transit time was observed using a 6% carmine red solution (in 0.5% methylcellulose). Individual rats without fasted beforehand were gavaged with the 10 μL/g of carmine solution at 9:00 a.m. The time from gavage to initial appearance of carmine in the feces was recorded as the gut transit time for that rat. On the other hand, rats were separately located into a sterile cage, and their cumulative fecal pellets of each rats within 60 min were recorded as corresponding defecation number.

*Metabolite derivatization and GC/MS condition*

The procedure of derivatization was performed as a previously method with minor modification (*62*). Briefly, A 60-µL volume of fecal extractive supernatant with 10 µL of ^13^C-L-valine (100 µg/mL) were dried and then mixed with 80 µL of methoxylamine solution (15 mg/mL in pyridine). The mixture was strongly vortexed for 1 min and incubated at 37 °C for 24 h. Subsequently, eighty microliters of BSTFA with 1% TMCS were added into the mixture and derivatized at 70 °C for 60 min, finally a 5-fold volume of n-hexane was added into the derivatives. Vortex and centrifugation were conducted prior to sample injection. Moreover, a gas chromatography coupled with a mass spectrum (GCMS-QP2010 systems, Shimaduzu Co., Tokyo, Japan) was applied for current metabolome analysis. Fecal derivatives were separated by a DB-5 MS fused-silica capillary column (30 m × 250 µm i.d.; Agilent J&W Scientific, Folsom, CA), chemically bonded with a 5% phenyl-95% methylpolysiloxane cross-linked stationary phase (0.25 µm film thickness). The instrumental conditions were set as following. A constant flow rate of carrier gas was set at 0.95 mL/min through the column under a splitless mode. A volume of 1-μL resulting derivatives was injected, and the solvent delay time was set to 6 min. The initial oven temperature was held at 60 °C for 2 min, ramped to 140 °C at a rate of 10 °C/min, to 240 °C at a rate of 4 °C/min, to 290 °C at a rate of 10 °C/min, and finally held at 290 °C for 8 min (total program time is 46 min). The temperatures of injector, transfer line, and electron impact (EI) ion source were set to 250 °C, 290 °C, and 230 °C, respectively. The electron energy was 70 eV, and mass data was collected in a full scan mode from m/z 50 to 600.

**Supplementary figures**


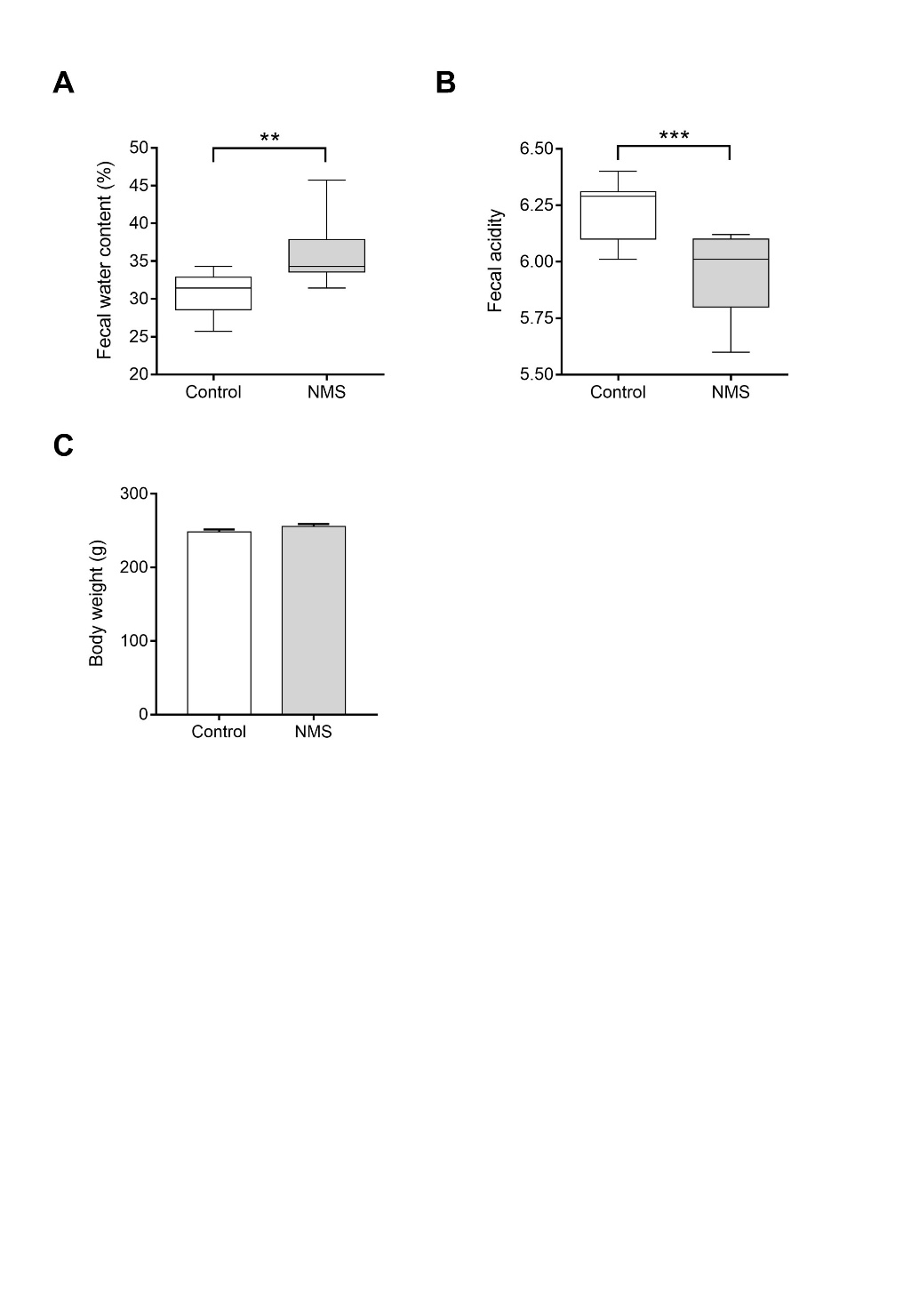


**Fig. S1**. Fecal characteristics (A, B) and body weight (C) in NMS rats and controls (n=8/group). The box plots are painted by medium with interquartile range (IQR). Bar chart is expressed as mean ± SEM. Statistical significance for comparison between both groups is defined as **, *p* < 0.01; ***, *p* < 0.005.

**
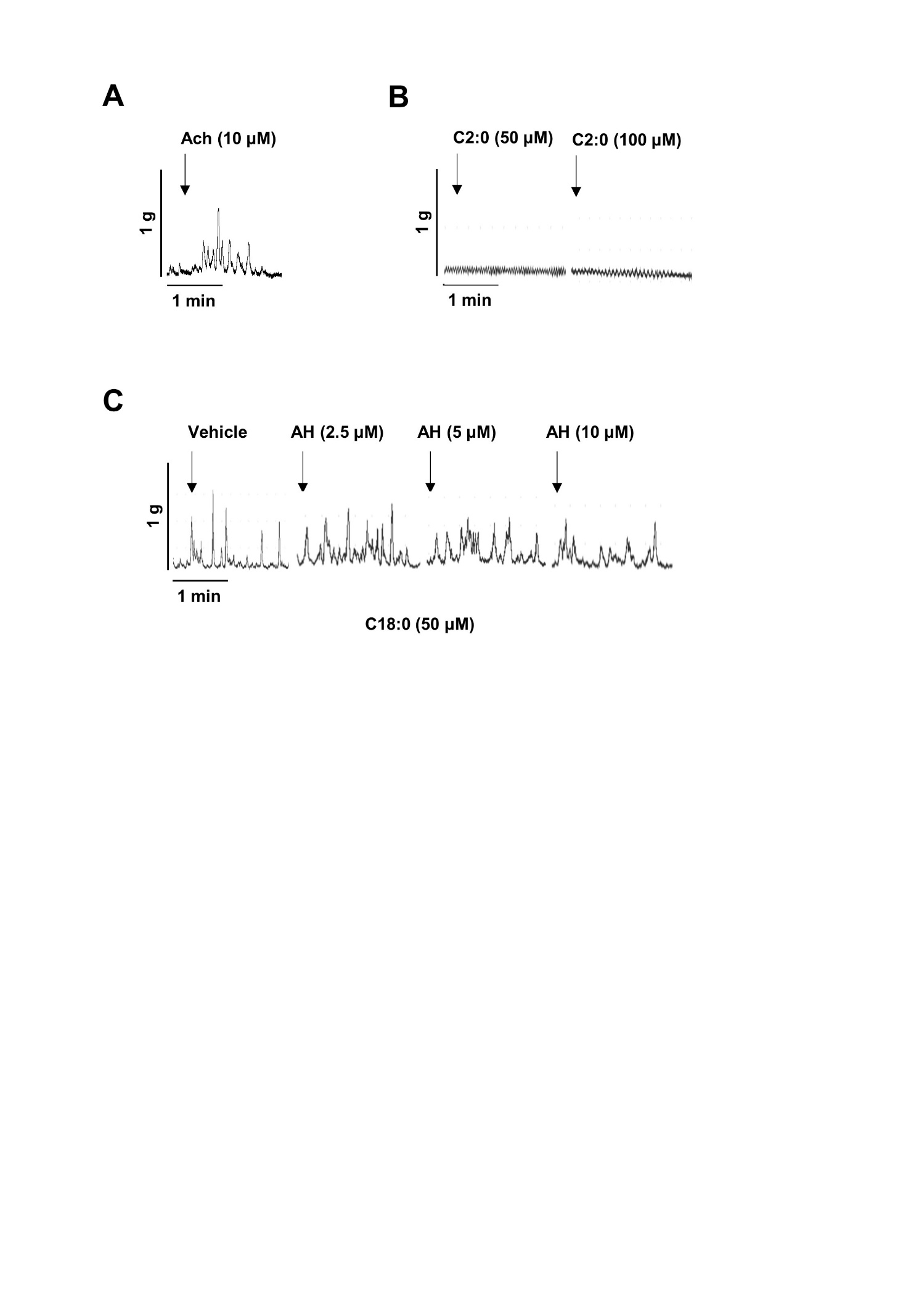
**

**Fig. S2**. Muscle amplitudes of rat isolated colonic segments with different treatment. (A, B) treatment of acetylcholine and C2:0 were used as positive and negative control for organ bath-based colonic motor tests, respectively. (C) The effects of GPR120 antagonist AH7614 on the colonic contraction induced by C18:0.


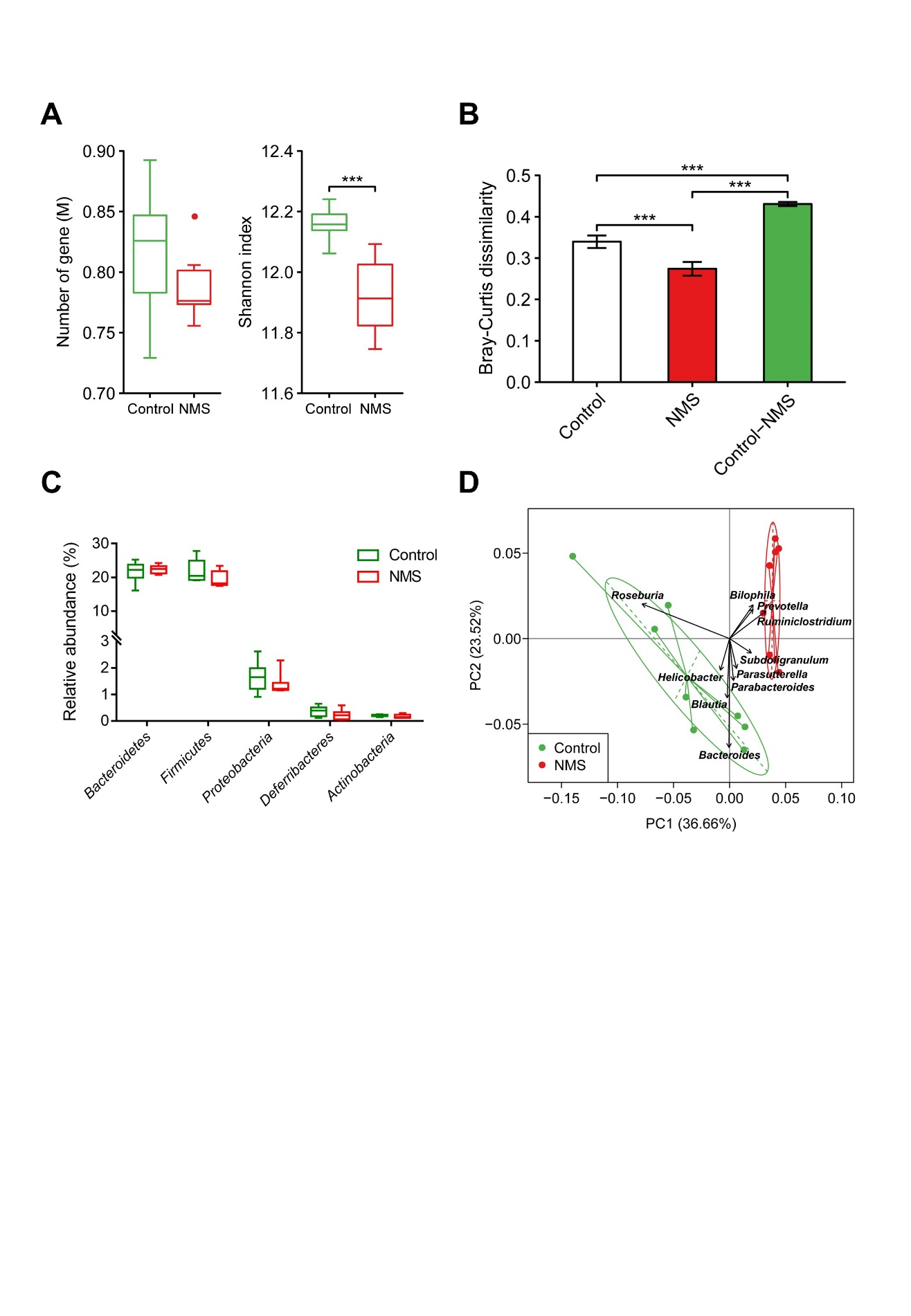


**Fig. S3**. Phylogenetic profiles of fecal microbiomes of NMS and control rats (n=8/group). (A) The level of microbial richness and α-diversity at gene level. (B) Bray-Curtis dissimilarity tested within and between both groups. (C) Dominant bacterial alteration between both group at the phylum level. (D) The biplot of bacterial variation between both groups at genus level. The first two principle components (PCs) was plotted with the percentage of variability explained indicated. Each point represents a sample. The box plots were painted by medium with IQR, bar chart was plotted using mean ± SEM, and the PCA biplot was formed based on the relative abundances of identified genera. Statistical significance is defined as ***, *p* < 0.005.


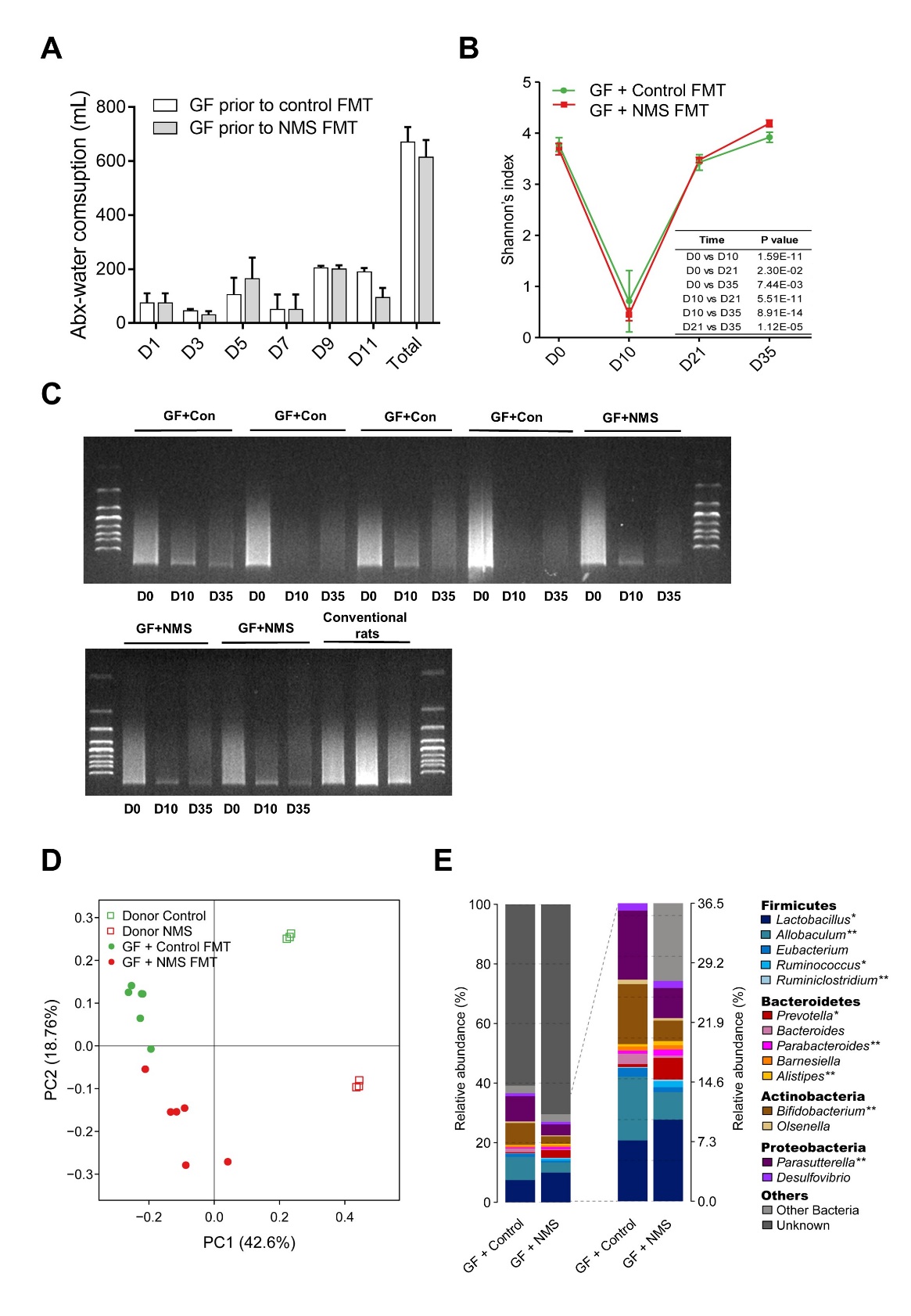


**Fig. S4**. Fecal microbial community in pseudo GF rats at duration of FMT experiment. (A) Consumption of ABX-contained water in GF models at duration of model establishment. (B) Dynamic changes of microbial diversities between both groups of recipients based on the Shannon index. (C) Agarose gel electrophoresis of microbial DNA extracts that was collected from GF rats on day 0, 10 and 35. The samples of conventional rats were extracted from feces of normal SD rats without ABX intervention. (D) Ecological β-diversities between recipients and donors based on Bray-Curtis metric. The first two principal coordinates (PCs) respectively explain 42.6 % and 18.76% of variation in the dataset. (E) The relative abundances of dominant bacterial genera in feces of recipients on Day 35. The significance is defined as *, *p* < 0.05; **, *p* < 0.01.

| **Table S1.** Fecal metabolites with significant difference between NMS rats and controls. | | | | | |
| --- | --- | --- | --- | --- | --- |
| ID | Rt (min) | Metabolite identification | FC (NMS vs. NH) | VIP value^*^ | *p* value |
| 1 | 7.0 | Lactic acid | 1.74 | 1.92 | 0.030 |
| 2 | 7.1 | Butyric acid | -1.35 | 1.77 | 0.010 |
| 3 | 8.0 | L-Alanine | -0.32 | 3.41 | 0.004 |
| 4 | 8.4 | Acetic acid | 0.65 | 1.07 | 0.041 |
| 5 | 8.7 | 2-Hydroxyisovaleric acid | 2.39 | 1.70 | 0.022 |
| 6 | 8.9 | Phosphoric acid | 0.55 | 1.01 | 0.004 |
| 7 | 9.2 | Glycerol | 0.61 | 1.33 | 0.022 |
| 8 | 9.8 | 2-Hydroxycaproic acid | 2.37 | 1.51 | 0.030 |
| 9 | 11.1 | Succinic acid | 1.71 | 2.23 | 0.034 |
| 10 | 11.2 | 3-Methylglutaconic acid | 1.14 | 1.11 | 0.024 |
| 11 | 18.3 | Lyxose | -1.00 | 3.45 | 0.002 |
| 12 | 19.3 | Ribitol | 2.61 | 1.74 | 0.044 |
| 13 | 21.6 | Citric acid | 4.06 | 1.06 | 0.049 |
| 14 | 22.4 | Tetradecanoic acid | 1.36 | 2.76 | 0.000 |
| 15 | 23.3 | Glucose | -1.58 | 2.37 | 0.014 |
| 16 | 24.4 | Glucaric acid | 1.82 | 1.41 | 0.017 |
| 17 | 24.7 | Pentadecanoic acid | 0.84 | 1.16 | 0.003 |
| 18 | 27.7 | Myo-Inositol | 2.68 | 2.72 | 0.021 |
| 19 | 29.1 | Heptadecanoic acid | 1.60 | 2.29 | 0.000 |
| 20 | 37.4 | 1-Monopalmitin | 0.18 | 1.08 | 0.017 |
| 21 | 39.2 | 2-Monostearin | 0.30 | 1.12 | 0.011 |
| *, VIP, the variable importance in the projection, which was obtained from a PLS-DA model on SIMCA-P software. | | | | | |
